# Microalgae from Cold Environments and Their Possible Biotechnological Applications

**DOI:** 10.3390/md21050292

**Published:** 2023-05-08

**Authors:** Eleonora Montuori, Maria Saggiomo, Chiara Lauritano

**Affiliations:** 1 Department of Ecosustainable Marine Biotechnology, Stazione Zoologica Anton Dohrn, Via Acton 55, 80133 Napoli, Italy; 2 Department of Chemical, Biological, Pharmaceutical and Environmental Sciences, University of Messina, Viale F. Stagno d’Alcontres 31, 98166 Messina, Italy; 3 Research Infrastructure for Marine Biological Resources Department, Stazione Zoologica, Via Acton 55, 80133 Napoli, Italy

**Keywords:** polar environments, microalgae, stressors, stress responses, bioactivity, biotechnology applications, carotenoids, lipids

## Abstract

Cold environments include deep ocean, alpine, and polar areas. Even if the cold conditions are harsh and extreme for certain habitats, various species have been adapted to survive in them. Microalgae are among the most abundant microbial communities which have adapted to live in low light, low temperature, and ice coverage conditions typical of cold environments by activating different stress-responsive strategies. These species have been shown to have bioactivities with possible exploitation capabilities for human applications. Even if they are less explored compared to species living in more accessible sites, various activities have been highlighted, such as antioxidant and anticancer activities. This review is focused on summarizing these bioactivities and discussing the possible exploitation of cold-adapted microalgae. Thanks to the possibility of mass cultivating algae in controlled photobioreactors, eco-sustainable exploitation is in fact possible by sampling a few microalgal cells without impacting the environment.

## 1. Introduction

Oceans cover more than 70% of the Earth’s surface, and are characterized by different environment typologies to which several organisms, sometimes specific ones, are adapted. Cold ecosystems, with a stable temperature below and/or at 0 °C, include deep ocean, alpine, and polar environments, areas where environmental conditions can become extreme. As the most abundant cold-adapted life-forms on earth, microorganisms dominate these environments in terms of species diversity and biomass [1]. Cold-adapted microorganisms include “psychrophilic” and “psychrotrophic” organisms; the former enjoy optimal growth at temperature ≤15 °C, while the latter are able to grow at temperatures below 15 °C but show a maximum optimal growth rate at >18 °C [2,3]. Thanks to their high-efficiency and energy-saving catalytic properties, cold-adapted microorganisms have become valuable natural resources with potential in various biological fields. The Arctic and Antarctic ecosystems are characterized by a high level of biological and genetic diversity, but are less studied compared to more accessible sites and are largely unexplored from the bioactivity point of view. This is mainly due to difficulties in sampling in such extreme habitats, e.g., ice coverage, as well as costs.

Environmental adaptations underlying physiological and molecular strategies are mechanisms allowing survival among cold-adapted microorganisms [4,5]. Adaptations to extreme, even hostile, temperatures can allow organisms to not only survive but to thrive and tolerate the extreme conditions of these natural environments. One of the major adaptations of metabolic function is the maintenance of membrane fluidity, which influences growth and photosynthesis at low temperatures [6,7,8]. Other general strategies include the ability to regulate the expression of structural enzymes, the expression of heat shock proteins, and the presence of compatible solutes.

Considering that temperature influences all the biochemical reactions of cells, the study of adaptations to temperature conditions is widespread. Recently, Lauritano et al. [4] reviewed physiological and molecular responses to the main environmental stressors of microalgae in polar marine environments. As reported in their review, low temperatures induced the maintenance of membrane fluidity in microalgae thanks to unsaturated fatty acids and the expression of cold shock/antifreeze proteins. In addition, depending on the concomitant variation of salinity and light intensity, variations in various lipid and pigment concentrations have been reported [4,9]. In particular, in the presence of high levels of ultraviolet radiation, an increase in photoprotective pigments, chlorophylls, and carotenoids has been found [10].

Furthermore, in the past decade cold-adapted microorganisms have attracted attention for their potential applications in biotechnology. Due to their metabolic activity at colder temperatures, these organisms or their derived products can be used in manufacturing or processing steps that require low temperatures [11,12]. Cold marine environments, including high latitude oceans, are dynamic across multiple spatial and temporal scales due to the complex interactions of ice formation and melting, ocean circulation and mixing, and terrestrial and atmospheric inputs.

The primary production of phytoplankton and sea ice algae in high latitude oceans is very high [13,14]. Microalgae are between the most abundant sea ice microbial communities that have adapted to live in extreme conditions and thrive within the distinct microhabitats created when sea ice forms and develops [15,16,17,18,19,20]. After becoming embedded in the ice, the algae must survive in extreme conditions of little or no light, high salinity, and cold temperatures within the sea ice brine. Their capability to live in different environments results in a portfolio of different taxonomic species and in the production of very different chemical mediators [21]. Microalgae have in fact been shown to be excellent producers of several bioactive molecules, and many studies have demonstrated that microalgal raw extracts, fractions, and/or pure molecules have biological activities with possible applications in the treatment of human pathologies, including antioxidant, anticancer, anti-microbial, and immunomodulatory activities [21].

**Figure 1:**
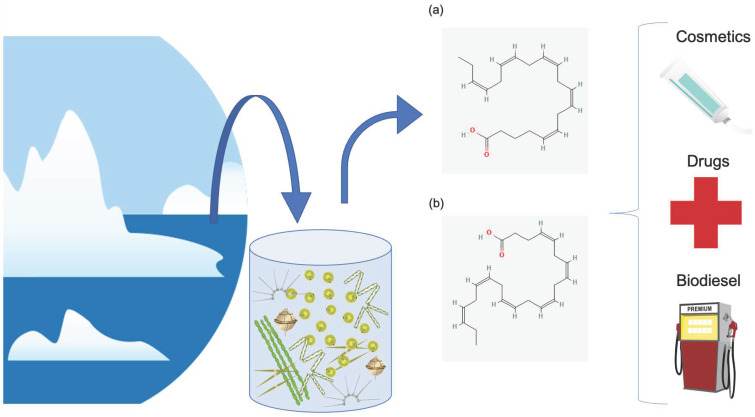
This figure summarizes how microalgal biodiversity from cold environments, as well as microalgae exposed to cold stress, may be sources of valuable products with different industrial applications, including ingredients for cosmetics, drugs, and biodiesel production. Chemical structures of (**a**) eicosapentaenoic acid (EPA) and (**b**) docosahexaenoic acid (DHA) were retrieved from the public database PubChem [25,26,27].

In addition to studies on cold adapted species, experiments in the laboratory have been performed to mimic natural conditions in order to study both their growth and physiological responses as well as to stress microalgae and induce the activation of metabolic pathways to obtain new activities and products. For instance, various researchers have applied the OSMAC (“one strain, multiple compounds”) approach, which means that different culturing conditions are used in order to stimulate the production of a high number of metabolites. Stressful culturing conditions may include variations in medium salinity, pH, temperature, nutrient concentrations, bubbling, and exposure to predators. Previous reviews have focused on bacterial and microalgal physiological and molecular responses in polar marine environments [4], omics approaches applied to polar microalgae linking the results with biochemical and physiological data ([22] and chapter by [23]), and extremophile microalgae, including various acidophilic/metallotolerant, alkaliphilic, endolithic/endophytic, halophilic, halotolerant, and cryophilic/psychrophilic microalgae, as well as their potential exploitation in the context of astrobiology [24]. The aim of the current review is to collect bioactivity information on microalgae living in cold environments, mainly polar ones, or exposed to cold stress in laboratory conditions, in order to discuss their activity properties and possible eco-sustainable exploitation for different applications (Figure 1).

## 2. Cold-Living Microalgae Bioactivities

The adaptation mechanisms of polar microalgae have evolved by developing ecological, physiological, and molecular defensive and adaptive strategies to withstand the harsh polar environment characterized by low temperatures, freeze-thaw cycles, desiccation, salinity, and high and variable photosynthetically active and ultraviolet radiation [28,29]. These adaptive strategies include the synthesis of a huge diversity of compounds originating from different metabolic pathways that protect them from stress caused by extreme environmental conditions [30]. Different compounds such as PUFAs [31,32], carotenoids [33,34], phenolic [34], UV-absorbing compounds [33], and exopolysaccharides [35] are overproduced by extreme environment microalgae [29], and have attracted biotechnological interest in various sectors [36].

### Antioxidant and Anticancer Activities

In 2018, Suh et al. [37] studied the anticancer and anti-inflammatory activity of the ethanol extract of freshwater microalga (green alga) *Micractinium* sp. (ETMI) on the human colon cancer cell line HCT116. The MTT (3-(4,5-dimethylthiazol-2-yl)-2,5-diphenyl-2H-tetrazolium bromide) assay and cell cycle assays showed significant suppression of cellular proliferation and induction of cell cycle arrest after treatment with 25, 50, and 100 μg/mL ETMI. In addition, ETMI at concentrations of 5, 10, 20, and 40 μg/mL showed anti-inflammatory activity by modulating inflammatory indicators such as cyclooxygenase (COX)-2, interleukin (IL)-6, inducible nitric oxide synthase (iNOS), tumor necrosis factor (TNF)-α, and nitric oxide (NO) in a dose-dependent manner [37]. Successively, Suh et al. [38] found that ethanol extract of the Arctic freshwater microalga (green alga) *Chloromonas reticulata* (ETCH) suppressed inflammation and carcinogenesis. ETCH showed anti-inflammatory activity at concentrations of 5, 10, 20, and 40 μg/mL by modulating major inflammatory markers such as COX-2, IL-6, iNOS, TNF-α, and NO production in a dose-dependent manner. Moreover, ETCH exhibited dose-dependent cytotoxic activity against HCT116 human colon cancer cells [38].

Recently, Ko et al. [39] demonstrated the bioactive properties of four Arctic algal extracts, the brown algae *Himantothallus grandifolius* and *Phaeurus antarcticus* and the red algae *Plocamium cartilagineum* and *Trematocarpus antarcticus* (former *Kallymenia antarctica*), on epithelial cells of human intestine and skin. Antarctic marine microalgal methanolic extracts increased cell viability of human colorectal adenocarcinoma cells Caco-2 and immortalized human keratinocyte HaCaT cells, protected cells against inflammatory stimulation, and increased the barrier integrity of cells damaged by lipopolysaccharide or ultraviolet radiation. The authors suggested that Antarctic marine microalgae extracts rich in fatty acids and lipids may, due to adaptation to the extreme polar environment, exert scavenging activity against specific radicals and intracellular reactive oxygen species (ROS) [39]. In 2023, León-Vaz et al. [34] explored the ability of nineteen strains of Nordic microalgae (green algae) to produce bioactive compounds: *Chlorococcum* sp. MC1, *Coelastrella* sp. 3–4, *Coelastrum astroideum* RW10, *Coelastrum microporum* FNY-1, *Desmodesmus opoliensis* SQ2,n *Desmodesmus* sp. RUC-2, *Desmodesmus* sp. 2–6, *Ettlia pseudoalveolaris* FNY-2, *Haematococcus pluvialis/lacustris* HP, *Monoraphidium* sp. B1–2, *Scenedesmus obliquus* 13–8, *Scenedesmus* sp. B2–2, *Scotiellopsis reticulata* UFA-2, *Chloroidium saccharophilum* (former *Chlorella saccharophila*) RNY, *Chlorella sorokiniana* 2–21–1, *Chlorella sorokiniana* B1–1, *Chlorella vulgaris* 13–1, *Chlorella vulgaris* LNY, *Micractinium* sp. P9–1). Chemical composition analysis showed that certain strains were able to produce high amounts of carotenoids and phenolic compounds. Because carotenoids and phenolic compounds are the two most relevant classes of antioxidant molecules in microalgae, León-Vaz et al. investigated the antioxidant activity of methanolic extracts of these nineteen Nordic microalgae with a 1,1-diphenyl-2-picrylhydrazyl (DPPH) radical scavenging assay [34]. The strains with the highest antioxidant activity were *Micractinium* sp. (P9-1), with an IC_50_ of 0.52 mg mL^−1^, *Desmodesmus opoliensis* SQ-2, with an IC_50_ of 0.58 mg mL^−1^, and *Scenedesmus* sp. B2-2, with an IC_50_ of 0.59 mg mL^−1^, while in *Chlorella* species the antioxidant capacity for scavenging DPPH radicals was lower [34].

In addition to studies analyzing single species, Ingebrigtsen et al. [40] investigated fifteen bulk plankton samples from three environmentally different areas (three samples from the coast of Finnmark, seven from the East of Hopen Island, and five from North West of Svalbard) for their antibacterial, anticancer, anti-diabetes, and antioxidant activities. After a freeze-drying step, the biomass composition present in each area was evaluated in terms of species characterization. Samples collected in the areas of Hopen East and northwest of Svalbard were completely or almost completely made up of phytoplankton. Each sample was fractionated using a gradient of MilliQ water, methanol, and acetone. Eight fractions per sample were obtained by this fractionation procedure and their bioactivity was further evaluated. Several of the fractions (from Fraction 4 to Fraction 7) showed cytotoxic activity against human melanoma A2058 cells. Only one of the fractions (Fraction 6 of an extract from northwest of Svalbard) was active in the cellular antioxidant assay (CAA), while in the cellular lipid peroxidation assay (CLPAA) there was detectable bioactivity in all fractions (1 to 8) in all areas with the greatest presence of phytoplankton, namely, Hopen East and northwest of Svalbard. Ingebrigtsen et al. compared the observed bioactivity with that of a mass-cultivated diatom, *Porosira glacialis*, identifying higher bioactivity in the field phytoplankton samples compared to the cultivated species *P. glacialis* [40]. Active marine microalgae are summarized in Table 1.

## 3. Cold Stress Exposure Experiments

Shukla et al. [46] evaluated the growth characteristics of the *Chlorella mirabilis* strain L-10 (isolated from deglaciated soil on King George Island, South Shetland, maritime Antarctic) in micro-scale mass cultivation systems under different nitrogen and carbon sources, and performed analyses of fatty acid contents. The biomass cultivation was performed in stable (indoor) and variable (outdoor) conditions. The indoor cultivation was performed in a closed cultivation chamber with an irradiation of 75 μmol photons m^−2^ s^−1^ at 15 °C. The outdoor condition was performed in a temperate region of the Czech Republic, Central Europe, selected as late winter or early spring (February–April), in order to mimic the temperature environments of the polar summer. The authors manipulated the growth conditions by treating the algae with various culture media rich in different nutrients. The fatty acid analyses in *Chlorella mirabilis* in the indoor experiment revealed increased production of linoleic acid in glycerol treatments (5% *v*/*v*). The authors concluded that *Chlorella mirabilis* could be considered a possible strain for linoleic acid production at low temperatures [46]. Linoleic acid is the most highly consumed polyunsaturated fatty acid (PUFA) in the human diet; it is an essential nutrient and is typically provided in enteral, parenteral, and infant clinical formulas [47].

In the same year, Boelen et al. [48] investigated the intraspecific variability in eicosapentaenoic acid (EPA) and docosahexaenoic acid (DHA) content in response to irradiance and temperature in the diatoms *Chaetoceros brevis* and *Thalassiosira weissflogii*, along with the interspecific variability of the same compounds in two polar diatoms (*Chaetoceros brevis*, *Thalassiosira weissflogii*) and three temperate microalgae (the green alga *Pyramimonas* sp., the haptophyta *Emiliania huxleyi*, and the raphidophyta *Fibrocapsa japonica*). Boelen et al. noted that higher relative and absolute levels of DHA were found in *Emiliania huxleyi* and *Pyramimonas* sp. cultivated at 3 °C and standard irradiation of 75 μmol photons m^−2^ s^−1^, while EPA levels were higher in *Fibrocapsa japonica*, *Chaetoceros brevis*, and *Emiliania huxleyi* cultivated at 16 °C and standard irradiation of 75 μmol photons m^−2^ s^−1^ [48]. EPA and DHA are well-known omega-3 fatty acids, and have been reported to play an important role for the immune system [49]. They have been shown to have anticancer [50], antibacterial, and antioxidant activities [51]. In particular, EPA has shown antibacterial activity against *Bacillus cereus* and *Staphylococcus aureus* [52] as well as anticancer properties against human A549 lung cancer cells and a human esophageal cancer cell line [53,54]. DHA has previously shown anticancer properties against breast (MDA-MB-231, MCF-7), pancreatic (MiaPaca-2), and colorectal (CaCo-2, SW-620) cancer cell lines [55]. Recently, Cheregi et al. [56] showed that the use of microalgal strains adapted to the local conditions in Nordic countries may be advantageous for biofuel and high-value compound production, especially for their high content of EPA and DHA. For instance, the diatom *Phaeodactylum tricornutum* (M28) from Norwegian fjords (at 10–15 °C) was able to accumulate 43% (dry weight) of lipids, with 7.33% EPA and 0.6% DHA. The diatom *Attheya septentrionalis* (M21; at 10–15 °C) from the Arctic region was able to accumulate 24% EPA.

Ingebrigtsen et al. [57] mass cultivated five Northern diatoms in large photobioreactors under three different culture condition and two irradiance and two temperature regimes (low irradiance: 30, 35, 55, 68, 76, 80, and 93 μmol photons m^−2^ s^−1^; high irradiance: 93, 115, 130, 135, and 160 μmol photons m^−2^ s^−1^; low temperature: 3.3, 4.3, 4.5, 5, and 5.6 °C; high temperature: 6.5, 6.6, 7.1, 8.5, 8.7, and 9 °C) to improve the production of secondary metabolites and test their bioactivities. The five diatom species, collected in the Barents Sea and along the coast of Northern Norway, were *Attheya longicornis*, *Chaetoceros furcellatus*, *Chaetoceros socialis*, *Porosira glacialis*, and *Skeletonema marinoi*. Depending on the growth conditions, chemical extracts (80% aqueous methanol) of several microalgae showed bioactive fractions with anticancer activity against human melanoma A2058 cells, immunomodulatory activity on human monocyte cells THP-1 with inhibition of TNFα, anti-diabetes activity, and antioxidant and/or antibacterial (against *Enterococcus faecalis*, *Staphylococcus aureus*, *Escherichia coli* and *Pseudomonas aeruginosa*) properties. *Attheya longicornis* was the only microalga which showed anticancer, anti-diabetes, antioxidant, immunomodulatory, and antibacterial activities in all three (4.3 °C and 76 μmol photons m^−2^ s^−1^; 9 °C and 30 μmol photons m^−2^ s^−1^; 9 °C and 130 μmol photons m^−2^ s^−1^) culturing conditions. *Chaetoceros furcellatus* showed anti-diabetes activity in all culturing conditions and anticancer activity only when cultivated within a range of low temperature and high irradiance (5 °C and 130 μmol photons m^−2^ s^−1^, respectively). *Chaetoceros socialis* cultured in high temperature and low light conditions (6.6 °C and 35 μmol photons m^−2^ s^−1^) showed anticancer and anti-diabetes activity, while two fractions from *C. socialis* extract cultivated at low temperature and high irradiance (4.5 °C and 130 μmol photons m^−2^ s^−1^) showed antioxidant activity. *Porosira glacialis* was active against cancer cells in both low irradiance (93, 80 μmol photons m^−2^ s^−1^) conditions of culturing and with high and low temperatures (6.5 °C and 80 μmol photons m^−2^ s^−1^). Two fractions of the extract of *P. glacialis* cultured in low temperature and low light conditions (3.3 °C and 93 μmol photons m^−2^ s^−1^) showed immunomodulatory and antioxidant activities, while the extract of the alga cultivated in high temperature and high light (7.1 °C and 135 μmol photons m^−2^ s^−1^) showed anti-diabetes activity. *Skeletonema marinoi* showed antioxidant activities in all three (5.6 °C and 115 μmol photons m^−2^ s^−1^; 8.5 °C and 55 μmol photons m^−2^ s^−1^; 8.7 °C and 93 μmol photons m^−2^ s^−1^) culturing conditions, and was active against cancer cells only when cultured at low temperature and high irradiance [57].

In 2017, El-Sheekh et al. [58] studied the effects of different temperatures (15, 20, 25, 30, 35, and 40 °C) on the growth, lipid productivity, and fatty acid profiles of the green alga *Scenedesmus acutus* as a possible feedstock for biodiesel production. The highest fatty acid content was observed at low temperature (15 °C). With increased temperatures, the fatty acid content decreased; hence, the authors suggested culturing *S. acutus* at low temperatures for biodiesel production purposes.

In the same year, Demirel et al. [59] determined the effects of three different culture media: Bold’s Basal Medium (BBM), Blue-Green medium (BG-11), and RD medium, along with two regimes of temperature values (22 and 28 °C), on the growth rate of *Desmodesmus protuberans*, and investigated the antioxidant and antimicrobial bioactivity of the crude extracts. The crude extract of *Desmodesmus protuberans* grown with BBM medium at 28 °C showed major antibacterial activities, inhibiting the growth of *Candida albicans*, while with the RD medium at 22 °C it showed major antioxidant activity [59]. Menegol et al. [60] studied the effects of temperatures of 22, 27, and 32 °C along with different sodium nitrate concentrations of 12, 24, 36, 48, or 60 mg L^−1^ on the chlorophyta *Heterochlorella luteoviridis. H. luteoviridis* was shown to be a great source of xanthophylls, carotenes, and polyunsaturated fatty acids. In particular, low temperatures improved the biosynthesis of ω3-type fatty acids by lowering the ω6:ω3 ratio [60].

Similarly, Stirk et al. [61], exposed six green microalgae, *Chlorococcum ellipsoideum*, *Gyoerffyana humicola*, *Nautococcus mamillatus*, *Acutodesmus acuminatus*, *Protococcus viridis* and *Chlorella vulgaris*, to salt and low temperature stress by the addition of 36 g L^−1^ NaCl and transfer from 25 °C to 15 °C. They analyzed and quantified changes in endogenous brassinosteroid content. Exposure for 30 min at 36 g L^−1^ NaCl defined an increase of brassinosteroid content in all six species compared with the corresponding untreated cultures. Only three species of microalgae, *Chlorococcum ellipsoideum*, *Acutodesmus acuminatus*, and *Protococcus viridis*, showed an increase in cell division when the temperature was lowered from 25 to 15 °C; on the contrary, salt stress had a negative effect on cell division [61]. As the six microalgae strains analyzed in this study were part of a previous study in which endogenous auxins, cytokinins, gibberellins, and brassinosterides were detected and quantified in four-day cultures [62,63], the authors suggested that it is likely that the rapid increase in endogenous brassinosterides in response to salt stress would have been accompanied by changes in the concentrations of other hormones as well.

Recently, Zaho et al. demonstrated lipid accumulation in the diatom *Phaeodactylum tricornutum*, the green alga *Chlorella vulgaris*, and the brown alga *Nannochloropsis* sp. grown in low temperature (15 °C) conditions. In this work, Zaho et al. determined the relative lipids and unsaturated fatty acid content with Fourier transform infrared (FTIR) spectroscopy [64]. In 2004, Jiang et al. showed that the production of eicosapentaenoic acid (EPA) and polyunsaturated fatty acids (PUFA) from the marine diatom *Pheodactylum tricornutum* increased following the induction of temperature stress (from 25 to 10 °C). The highest yield per dry mass unit was 4.9% for PUFA (dry mass: 12.4 mg L^−1^) and 2.6% for EPA (dry mass: 6.6 mg L^−1^) when the temperature was shifted from 25 to 10 mid-hour [65]. In 2022, Fierli et al. [66] further studied lipogenesis and carotenogenesis in the diatom *Pheodactylum tricornutum* exposed to a cold temperature shock (from 20 to 10 °C) with addition of NaCl salt (5 mg mL^−1^) and the phytohormone abscisic acid (4 mg L^−1^). At 24 h and 5 days from stress induction with low temperature, salt, and the phytohormone abscisic acid, Fierli et al. analyzed the expression of key genes such as diacylglycerol O-acyltransferase 1 (DAGT), zeaxanthin epoxidase (ZEP3), and coding enzymes involved in the synthesis of lipids and carotenoids, demonstrating that cold temperature influenced lipid production, increasing it by 22%. At the same time, they noted an increase in EPA of 22% and fucoxanthin of 33% [66]. Lipids can have applications in the nutraceutical and cosmeceutical fields, while fucoxanthin has been shown to have anticancer [67] and anti-obesity activities [68]. Active marine microalgae are summarized in Table 2.

## 4. Conclusions

Many microalgae have been reported to live in cold extreme environments. While certain algal cells can form resting spores/cysts, many polar algae appear to remain in a vegetative state during winter [69]. This review shows that cold-adapted species are able to exert bioactivities for different human applications. In particular, anticancer (mainly melanoma cells), immunomodulatory, and antioxidant activities have been reported.

Microalgae are photosynthetic unicellular organisms that, in certain cases, can be massively cultivated under controlled conditions in photobioreactors in an eco-friendly and eco-sustainable way, thereby reducing destructive collection practices applied to sampling huge numbers of individuals of certain macro-organisms. This means that a few sampled cells are sufficient, and that culturing and successive studies can be performed in laboratory conditions without impacting natural environments. In addition, recent advances in -omics technologies, such as genomics, metagenomics, transcriptomics, metatranscriptomics, proteomics, and metabolomics, can be applied to microalgae to perform in silico analysis of metabolites of interest (e.g., [70]). In particular, these approaches have helped to shed light on biosynthetic pathways that can be involved in the synthesis of compounds with ecological functions (e.g., chemical mediators, communication, and defense), as well as possible biotechnological applications (e.g., pharmaceutical, nutraceutical, cosmeceutical, and biofuel applications).

Among the most active species, for anticancer activity there is *Botrydiopsidaceae* sp. (ETBO; active at 6.25 µg/mL); for immunomodulatory activity *Chloromonas reticulata* (ETCH) and *Micractium* sp. (ETMI) (active at 5 µg/mL); and for antioxidant activity, *Micractinium* sp. (P9-1), with an IC_50_ of 0.52 mg mL^−1^, *Desmodesmus opoliensis* SQ-2, with an IC_50_ of 0.58 mg mL^−1^, and *Scenedesmus* sp. B2-2, with an IC_50_ of 0.59 mg mL^−1^. Considering that these are raw extracts, not pure compounds, successive purification of bioactive metabolites is necessary in order to compare them to the marine pharmaceuticals already on the market, many of which are active at the level of 0.6–7 ng/mL (see Table 3 in Martinez et al. [71]).

Algae have been found to be excellent sources of various compound categories, such as fatty acids, peptides, carbohydrates, vitamins, polyphenols, and pigments [21,72]. Among the most active reported compounds in cold microalgal species are the omega-3 fatty acids EPA and DHA. Humans produce very low rates of EPA and DHA, while these are fundamental nutrients in our diet. EPA and DHA are hormonal precursors, play an important role in the immune system [49], and have been shown to have anticancer [50] and antioxidant activities [51], which is why they are of great economic interest. It has been hypothesized that microalgae living in polar regions have higher levels of PUFAs, as in addition to playing a role in the functioning of the thylakoid membrane, they serve to contain the fluidity of the cell membrane [48,73]. For this reason, polar microalgae adapted to cold and showing high growth rates in low-temperature and low-irradiance conditions could be used as key species for mass production of EPA and DHA.

In addition to production of bioactive compounds, cold-adapted microalgal species have shown promising bioremediation properties [74,75]. For example, Gojkovic and collaborators tested eight microalgal species from Northern Sweden and the culture collection strain *Scenedesmus obliquus* RISE (UTEX 417) to remove nineteen pharmaceuticals from growth medium in bioreactors. Lipophilic compounds such as Biperiden, Trihexyphenidyl, Clomipramine, and Amitriptyline were removed efficiently from the culture medium (>70% in average within 2 days). Interestingly, *Chlorella vulgaris* 13-1 and *Chlorella saccharophila* RNY were highly efficient in removing all nineteen pharmaceuticals from the growth medium within 12 days.

Warming oceans, changing ocean circulation, decreasing ice cover, and increasing freshwater inputs are all expected to impact polar ecosystems in the future. To date, while many efforts have been made to measure changes in phytoplankton biomass and in community composition in cold environments, less attention has been paid to the physiological responses and metabolites they may produce, which require further studies. Physiological changes in microalgae influence their growth rate, fitness, nutritional quality, and compound/secondary metabolite production [76], and further investigations can shed light on new details regarding the influence on primary production rates, community composition, cold environment ecology, and possible biotechnological exploitation.

## Figures and Tables

**Table 1:** This table reports bioactivities reported for microalgae living in cold environments, with the sampling location, performed assays, and tested concentration. DPPH stands for 1,1-diphenyl-2-picrylhydrazyl; ELISA for Enzyme-linked immunosorbent assay; FITC for fluorescein isothiocyanate; MTS for 3-(4,5-dimethylthiazol-2-yl)-5-(3-carboxymethoxyphenyl)-2-(4-sulfophenyl)-2H-tetrazolium; MTT for 3-(4,5-dimethylthiazol-2-yl)-2,5-diphenyl-2H-tetrazolium bromide; and ROS for reactive oxygen species.

Microalga	Identified Compounds	Activity	Assay, Cell Line, Concentration	Sampling Location	Reference
*Chloromonas reticulata*	-	Anti-inflammation, anti-carcinogenesis	MTS assay was performed to determine the cytotoxic effect of ETCH ELISA was performed to determine the amounts of cytokines in the cell culture after 18 h of treatment with different concentrations of ETCH (5, 10, 20, 40 µg/mL); Cell lines: RAW 264.7, HCT116	King Sejong Station (Average temperature −1.8 °C, according to https://www.kopri.re.kr/eng/html/infra/03010101.html; accessed on 21 April 2023)	[38]
*Desmodesmus opoliensis* SQ-2	Neoxanthin, violaxanthin, antheraxanthin, lutein, c-lutein, astaxanthin, chlorophyll b, chlorophyll a,β,ε-carotene and ββ-carotene	Antioxidant assay	DPPH radical scavenging assay was performed to test the antioxidant activity. Extracts were tested at different concentrations: 1, 0.8, 0.6, 0.4 and 0.2 mg mL^−1^; IC_50_ of 0.58 mg mL^−1^	Nordic microalgae (Summer 15 °C, Winter −4 °C)	[34]
*Himantothallus grandifolius*	9-Octadecenoic acid, octadecanoic acid, 9,12-octadecadienoic acid, essential fat-soluble vitamin E	Increased cell viability, intracellular ROS scavenging, anti-inflammatory activity	To determine the cytotoxicity, Caco-2 cells and HaCaT cells were incubated for 18 h with 1 µg/mL of extract; Intracellular ROS Scavenging	Antarctic region (Winter −1.7 °C, Summer 3.0 °C) [41]	[39]
*Trematocarpus antarticus* (former *Kallymenia antarctica*) [42,43]	9-Octadecenoic acid, cholesterol, heptadecane	Increased cell viability, intracellular ROS scavenging, anti-inflammatory activity	To determine the cytotoxicity, Caco-2 cells and HaCaT cells were incubated for 18 h with 1 µg/mL of extract	Antarctic region (Winter −1.7 °C, Summer 3.0 °C)	[39]
*Micractinium* sp.	-	Anti-inflammatory, anticancer	MTT assay and Cell cycle assay after 24 h of treatment with ETMI at different concentrations (25, 50, and 100 μg/mL); To evaluate the effect of ETMI on proinflammatory cytokine expression, RAW 264.7 macrophages were treated with different concentrations of ETMI (5, 10, 20, 40 μg/mL) for 1 h and then stimulated with 1 mg mL^−1^ LPS for 24 h; Cell lines: RAW 264.7, HCT116;	King Sejong Station (Average temperature −1.8 °C)	[37]
*Micractinium* sp. (P9-1)	Neoxanthin, violaxanthin, antheraxanthin, lutein, c-lutein, astaxanthin, chlorophyll b, chlorophyll a,β,ε-carotene and ββ-carotene	Antioxidant assay	DPPH radical scavenging assay was performed to test the antioxidant activity; Extracts were tested at different concentrations: 1, 0.8, 0.6, 0.4 and 0.2 mg mL^−1^; IC_50_ of 0.51 mg mL^−1^	Nordic microalgae (Summer 15 °C, Winter −4 °C)	[34]
*Phaeurus antarcticus*	octadecanoic acid, 9,12-octadecadienoic acid, essential fat-soluble vitamin E	Increased cell viability, intracellular ROS scavenging, anti-inflammatory activity	To determine the cytotoxicity, Caco-2 and HaCaT cells were incubated for 18 h with 1 µg/mL of extract	Antarctic region (Winter −1.7 °C, Summer 3.0 °C) [44]	[39]
*Plocamium cartilagineum*	tetradecanoic acid, neophytadiene, heptadecane	Increased cell viability, intracellular ROS scavenging, anti-inflammatory activity	To determine the cytotoxicity, Caco-2 and HaCaT cells were incubated for 18 h with 1 µg/mL of extract	Antarctic region (Winter −1.7 °C, Summer 3.0 °C) [45]	[39]
*Scenedesmus* sp. B2-2	Neoxanthin, violaxanthin, antheraxanthin, lutein, c-lutein, astaxanthin, chlorophyll b, chlorophyll a,β,ε-carotene and ββ-carotene	Antioxidant assay	DPPH radical scavenging assay was performed to test the antioxidant activity; Extracts were tested at different concentrations: 1, 0.8, 0.6, 0.4 and 0.2 mg mL^−1^. IC_50_ of 0.59 mg mL^−1^	Nordic microalgae (Summer 15 °C, Winter −4 °C)	[34]

**Table 2:** This table shows bioactivities reported for microalgae exposed to cold stress along with the experiments, performed assays, and tested concentrations. BBM stands for Bold’s Basal Medium; BG-11 for Blue-Green medium; DHA for docosahexaenoic acid; ELISA for Enzyme-linked immunosorbent assay; EPA for eicosapentaenoic acid; FRAP for Ferric Reducing Antioxidant Power; FTIR for Fourier-transform infrared spectroscopy; MTS for 3-(4,5-dimethylthiazol-2-yl)-5-(3-carboxymethoxyphenyl)-2-(4-sulfophenyl)-2H-tetrazolium; PTP1B for Phosphatase1B Inhibition Assay; and TBARS for thiobarbituric acid reactive substances.

Microalga	Activity	Assay, Cell Line, Concentration	Cold Stress Exposure Conditions	Reference
*Acutodesmus acuminatus*	-	Evaluation of endogenous brassinosteroid content	Exposure at 36 g L^−1^ NaCl and low temperature stress from 25 °C to 15 °C	[61]
*Attheya longicornis*	Anticancer, antibacterial, immunomodulatory, anti-diabetes	Cell line: A2058, THP-1; Assay: FRAP antioxidant assay, antibacterial assays, enzymatic PTP1B diabetes assay, MTS cell viability assay, ELISA immunomodulation assay	Low temperature exposure at 4.3 °C and low irradiance of 76 μmol photons m^−2^ s^−1^. High temperature exposure at 9 °C and low irradiance of 30 μmol photons m^−2^ s^−1^. High temperature exposure at 9 °C and high irradiance of 130 μmol photons m^−2^ s^−1^.	[57]
*Chaetoceros brevis*	-	Variability of EPA and DHA content analyses	Cultivated at 3 °C and 7 °C with an irradiance of 10, 25, 75 and 150 μmol photons m^−2^ s^−1^	[48]
*Chaetoceros furcellatus*	Anti-diabetes, anticancer	Cell line: A2058, THP-1; Assay: FRAP antioxidant assay, antibacterial assays, enzymatic PTP1B diabetes assay, MTS cell viability assay, ELISA immunomodulation assay	Low temperature exposure at 4.3 °C and low irradiance of 30 μmol photons m^−2^ s^−1^. Low temperature exposure at 5 °C and high irradiance of 130 μmol photons m^−2^ s^−1^. High temperature exposure at 8.5 °C and low irradiance of 68 μmol photons m^−2^ s^−1^.	[57]
*Chaetoceros socialis*	Antioxidant, anti-diabetes	Cell line: A2058, THP-1; Assay: FRAP antioxidant assay, antibacterial assays, enzymatic PTP1B diabetes assay, MTS cell viability assay, ELISA immunomodulation assay	Low temperature exposure at 4.3 °C and high irradiance of 130 μmol photons m^−2^ s^−1^. High temperature exposure at 6.6 °C and low irradiance of 35 μmol photons m^−2^ s^−1^.	[57]
*Chlorella mirabilis* L-10	-	Fatty acid analyses in biomass cultivation in stable (in-door) and variable (out-door) conditions	In-door cultivation performed at 15 °C with an irradiance of 75 µmol m^−2^ s^−1^. Out-door cultivation performed at the night-day temperature ranged from −6.6 to 17.5 °C and irradiance ranged from 0 to 2300 mol m^−2^ s^−1^.	[46]
*Chlorella vulgaris*	-	Evaluation of endogenous brassinosteroid content	Exposure at 36 g L^−1^ NaCl and low temperature stress from 25 to 15 °C	[61]
*Chlorella vulgaris*	-	FTIR spectroscopy	Cold temperature of 15 °C	[64]
*Chlorococcum ellipsoideum*	-	Evaluation of endogenous brassinosteroid content	Exposure at 36 g L^−1^ NaCl and low temperature stress from 25 to 15 °C	[61]
*Desmodesmus protuberans*	Antioxidant, antibacterial	DPPH Assay, Antimicrobial Activity Assay	Three different culture media: BBM, BG-11 and RD Two regimes of temperature: 22 and 28 °C Light intensity of 75 μmol m^−2^ s^−1^	[59]
*Emiliania huxleyi*	-	Variability of EPA and DHA content analyses	Cultivated at temperature of 16 °C with an irradiance of 75 μmol photons m^−2^ s^−1^	[48]
*Fibrocapsa japonica*	-	Variability of EPA and DHA content analyses	Cultivated at temperature of 16 °C with an irradiance of 75 μmol photons m^−2^ s^−1^	[48]
*Heterochlorella luteoviridis*	-	Biomass production and composition: carbohydrates, carotenoids and lipids	Different temperatures (22, 27 or 32 °C) and sodium nitrate concentrations (12, 24, 36, 48 or 60 mg L^−1^ of N^−^NO_3_)	[60]
*Nannochloropsis* sp.	-	FTIR spectroscopy	Cold temperature 15 °C	[64]
*Nautococcus mamillatus*	-	Evaluation of endogenous brassinosteroid content	Exposure at 36 g L^−1^ NaCl and low temperature stress from 25 to 15 °C	[61]
*Pheodactylum tricornutum*	-	Nile red fluorescence assay to evaluate lipid content; TBARS assay to evaluate lipids peroxidation; Real Time-PCR	Cold temperature shock from 20 to 10 °C NaCl salt 5 mg mL^−1^ Phytohormone abscisic acid (4 mg L^−1^)	[66]
*Pheodactylum tricornutum*	-	FTIR spectroscopy	Cold temperature of 15 °C	[64]
*Porosira glacialis*	Anticancer, anti-inflammatory, antioxidant, anti-diabetes	Cell line: A2058, THP-1 Assay: FRAP antioxidant assay, antibacterial assays, enzymatic PTP1B diabetes assay, MTS cell viability assay, ELISA immunomodulation assay	Low temperature exposure at 3.3 °C and high irradiance of 93 μmol photons m^−2^ s^−1^. Low temperature exposure at 4.5 °C and high irradiance of 140 μmol photons m^−2^ s^−1^. High temperature exposure at 6.5 °C and low irradiance of 80 μmol photons m^−2^ s^−1^. High temperature exposure at 7.1 °C and high irradiance of 135 μmol photons m^−2^ s^−1^.	[57]
*Protococcus viridis*	-	Evaluation of endogenous brassinosteroid content	Exposure at 36 g L^−1^ NaCl and low temperature stress from 25 to 15 °C	[61]
*Pyramimonas* sp.	-	Variability of EPA and DHA content analyses	Cultivated at temperature of 3 °C with an irradiance of 75 μmol photons m^−2^ s^−1^	[48]
*Scenedesmus acutus*	-	Lipid productivity and fatty acid profiles	Range of temperature of 15, 20, 25, 30, 35 and 40 °C	[58]
*Skeletonema marinoi*	Anticancer, antioxidant	Cell line: A2058, THP-1; Assay: FRAP antioxidant assay, antibacterial assays, enzymatic PTP1B diabetes assay, MTS cell viability assay, ELISA immunomodulation assay	Low temperature exposure at 5.6 °C and high irradiance of 115 μmol photons m^−2^ s^−1^. High temperature exposure at 8.5 °C and low irradiance of 55 μmol photons m^−2^ s^−1^. High temperature exposure at 8.7 °C and high irradiance of 93 μmol photons m^−2^ s^−1^.	[57]
*Thalassiosira weissflogii*	-	Variability of EPA and DHA content analyses	Cultivated at temperature of 16 °C and 20 °C with an irradiance of 10, 25, 75 and 150 μmol photons m^−2^ s^−1^	[48]

## Data Availability

Not applicable.
